# Spatiotemporal link between El Niño Southern Oscillation (ENSO), extreme heat, and thermal stress in the Asia–Pacific region

**DOI:** 10.1038/s41598-024-58288-0

**Published:** 2024-03-28

**Authors:** Jakob Eggeling, Chuansi Gao, Dong An, Raul Cruz-Cano, Hao He, Linus Zhang, Yu-Chun Wang, Amir Sapkota

**Affiliations:** 1 Aerosol and Climate Laboratory, Division of Ergonomics and Aerosol Technology, Department of Design Sciences, Faculty of Engineering (LTH), Lund University, Lund, Sweden; 2 Division of Water Resources Engineering, Faculty of Engineering (LTH), Lund University, Lund, Sweden; 3 Department of Epidemiology and Biostatistics, School of Public Health, Indiana University, Bloomington, IN 47405 USA; 4 Department of Atmospheric and Oceanic Science, University of Maryland, College Park, MD 20742 USA; 5 Department of Environmental Engineering, College of Engineering, Chung Yuan Christian University, 200 Chung-Pei Road, Zhongli, 320 Taiwan; 6 Department of Epidemiology and Biostatistics, School of Public Health, University of Maryland, College Park, MD 20742 USA

**Keywords:** Climate sciences, Climate change

## Abstract

Climate change is closely monitored and numerous studies reports increasing air temperature and weather extremes across the globe. As a direct consequence of the increase of global temperature, the increased heat stress is becoming a global threat to public health. While most climate change and epidemiological studies focus on air temperature to explain the increasing risks, heat strain can be predicted using comprehensive indices such as Universal Thermal Climate Index (UTCI). The Asia–Pacific region is prone to thermal stress and the high population densities in the region impose high health risk. This study evaluated the air temperature and UTCI trends between 1990 and 2019 and found significant increasing trends for air temperature for the whole region while the increases of UTCI are not as pronounced and mainly found in the northern part of the region. These results indicate that even though air temperature is increasing, the risks of heat stress when assessed using UTCI may be alleviated by other factors. The associations between El Niño Southern Oscillation (ENSO) and heat stress was evaluated on a seasonal level and the strongest regional responses were found during December-January (DJF) and March–May (MAM).

## Introduction

As reported by the IPCC Assessment Report 6^1,2^ and recent studies^3,4^, global trends of increasing temperatures are continuously observed which will have detrimental effects on public health. Extreme weather events such as heat waves are expected to increase with the increasing air temperature (Ta) which will increase the risk of thermal stress^1,5,6^. Thermal stress is caused by exposure to too hot or too cold environments that cause the human body strained physiological reactions, if left accounted for, they can cause severe consequence such as discomfort, reduced work capacity, heat related illnesses or even death. The increase in Ta has the effect of increasing evaporation rate from the earth surface and the water vapor holding capacity of the air^7^ that increases water vapor pressure and exacerbates heat stress. The eastern Asia–Pacific region was identified to be the most exposed to deadly climatic conditions in a study that evaluated extreme heat^8^ where living and working conditions are already strained^9,10^. Dunne et al. (2011) forecast that the working conditions will become too severe to maintain normal production during the hottest month of the year^11^, which is around April–October in the Asia–Pacific region. Ta is often used to define extreme heat and climate change and has merit due to its simplicity and long track record, however, it lacks other relevant information that affects human body heat balance and thermal stress^12^. The human body heat exchange with the environment is also affected by climatic factors like air humidity, wind speed, radiant temperature, and the personal factors such as metabolic rate and clothing insulation and evaporative resistance, etc. The most effective avenue of heat stress relief is by sweating where evaporation cools the skin by lowering the average energy of the remaining sweat. The evaporation rate is dependent on the water vapor pressure gradient between the skin surface and the air which is highly influenced by the hydrological cycle. By increasing the wind speed, the air next to skin being saturated by evaporated sweat is replaced by less saturated air which increases the gradient. The radiant temperature commonly exacerbates heat stress by continuously heating up the body where the hydrological cycle may alleviate this external forcing by cloud formation when supersaturation occur in the atmosphere. There exists an extensive library of thermal indices that are derived from a combination of these relevant factors such as Wet Bulb Globe Temperature (WBGT)^13^ or Humidex^14^, or in full in human thermal models such as Predicted Heat Strain^15^ to comprehensively assess thermal exposure and physiological responses. A review of thermal indices was undertaken by de Freitas and Grigorieva (2015), in their study most existing available indices are cataloged^16^ and later followed up by Ioannou et al. (2022) who further compared available indices and found WBGT and the Universal Thermal Climate Index (UTCI) to be the most appropriate indices to predict thermal stress for outdoor workers^17^.

UTCI utilizes totally six variables (Ta, humidity, wind speed, radiant temperature, activity and clothing) and calculates an equivalent temperature index that better reflects the thermal exposure compared to the reference condition^18,19^. UTCI development was started by a commission from the International Society of Biometeorology and further joint development with European COST Action 730. The UTCI model has been applied in increasingly more studies throughout the world assessing the spatiotemporal patterns and changes of thermal stress including cold and heat stress^20–29^. Still, the Asia–Pacific region as a whole is not well studied when applying UTCI. Zeng et al. (2020) found an increasing UTCI trend in the China-Pakistan Economic Corridor during 1979–2018^30^, Jacobs et al. (2019) observed UTCI in three major cities in South Asia during 2006 (SAS) ^31^ while Ullah et al. (2022) looked at the SAS region and found primarily increasing trends during 1981–2019^28^.

Reanalysis data has become more readily available and has been increasingly utilized in climate research related to human thermal stress evaluations^32–34^. The European Centre for Medium-Range Weather Forecasts (ECMWF) has created the dataset ERA5-HEAT (Human thermal comfort) evaluating UTCI based on the environmental data from their ERA5 (5th generation ECMWF global reanalyzes) product^35^. Reanalysis data has also been used to evaluate the effects of teleconnections on regional and local climate trends as they have proven to correlate with extreme weather and changes in weather trends^36–39^.

The climate in the Asia–Pacific region is greatly affected by the East Asian monsoon and teleconnections such as El Niño Southern Oscillation (ENSO)^40–42^, the Indian Ocean Dipole (IOD)^43^, the North Atlantic Oscillation (NAO)^44^ and the Pacific Decadal Oscillation (PDO)^45^. The hydrological cycles on Earth are driven by solar radiation from the sun forming pressure cells which force air movement and vast amounts of energy to be displaced, the teleconnections are identified systems driven by these cycles. Depending on the state and location of the pressure cells, regional climate will experience variations of wind speed, precipitation characteristics and temperature profiles. As these variations are greatly influencing UTCI, the thermal stress experienced by the populations in the regional climate will be dependent on the state of the teleconnections such as ENSO. Existing research has well documented the impacts of these teleconnections with local and regional climate with focus on Ta and precipitation^39^. Heat waves in China have can be affected by ENSO where low pressure centers in the Pacific and Indian Ocean are induced by El Niño as well as intensification and westward extension of the subtropical high-pressure center which leads to favorable conditions for heat waves in southern China^46^. When evaluating extreme precipitation indices, it has been found that these are more affected by the local topography compared to the regional topography which has a bigger effect on hydrological systems^47^. Recently studies have applied UTCI when evaluating the effects of climate change on human thermal stress in relation with ENSO^28,29^. Sea surface temperature (SST) in specific regions is monitored to predict ENSO events with a 6–12 month lead time, this promotes its suitability for incorporation into sub-seasonal to seasonal heat health warning systems (HWS). ENSO oscillates around the warm phase El Niño where warm water pools in the eastern Pacific Ocean and the cold phase La Niña where an enhanced upwelling of cold water is seen in the eastern Pacific Ocean. The neutral phase of ENSO resembles a weaker La Niña phase where the trade winds push warm surface water to the western Pacific Ocean and upwelling of cold water occur in the eastern Pacific Ocean^40^. This study aims to identify trends of extreme heat over a large region in the Asia–Pacific region and evaluate the impact of ENSO on spatiotemporal patterns of the seasonal climate variations. The seasons defined in this study are MAM (March, April, May), JJA (June, July, August), SON (September, October, November), and DJF (December, January, February). They are referenced to as the boreal seasons spring (MAM), summer (JJA), autumn (SON) and winter (DJF). Based on the climate change depicted in the most recent IPCC assessment report^1^ and studies focusing on UTCI in parts of the region^28,30,31^, we hypothesize that UTCI and Ta trends are increasing throughout the region. As the hydrological cycle is shown to be impacted by ENSO^39,40,42,48^, we expect to see a correlation between ENSO and UTCI. By further cataloging the spatial trends of thermal stress using UTCI and the effect of ENSO on local seasonal climate, future policymaking, and HWS may alleviate future climate hazards.

## Results

### Spatial analyses of UTCI and Ta trends

**Figure 1:**
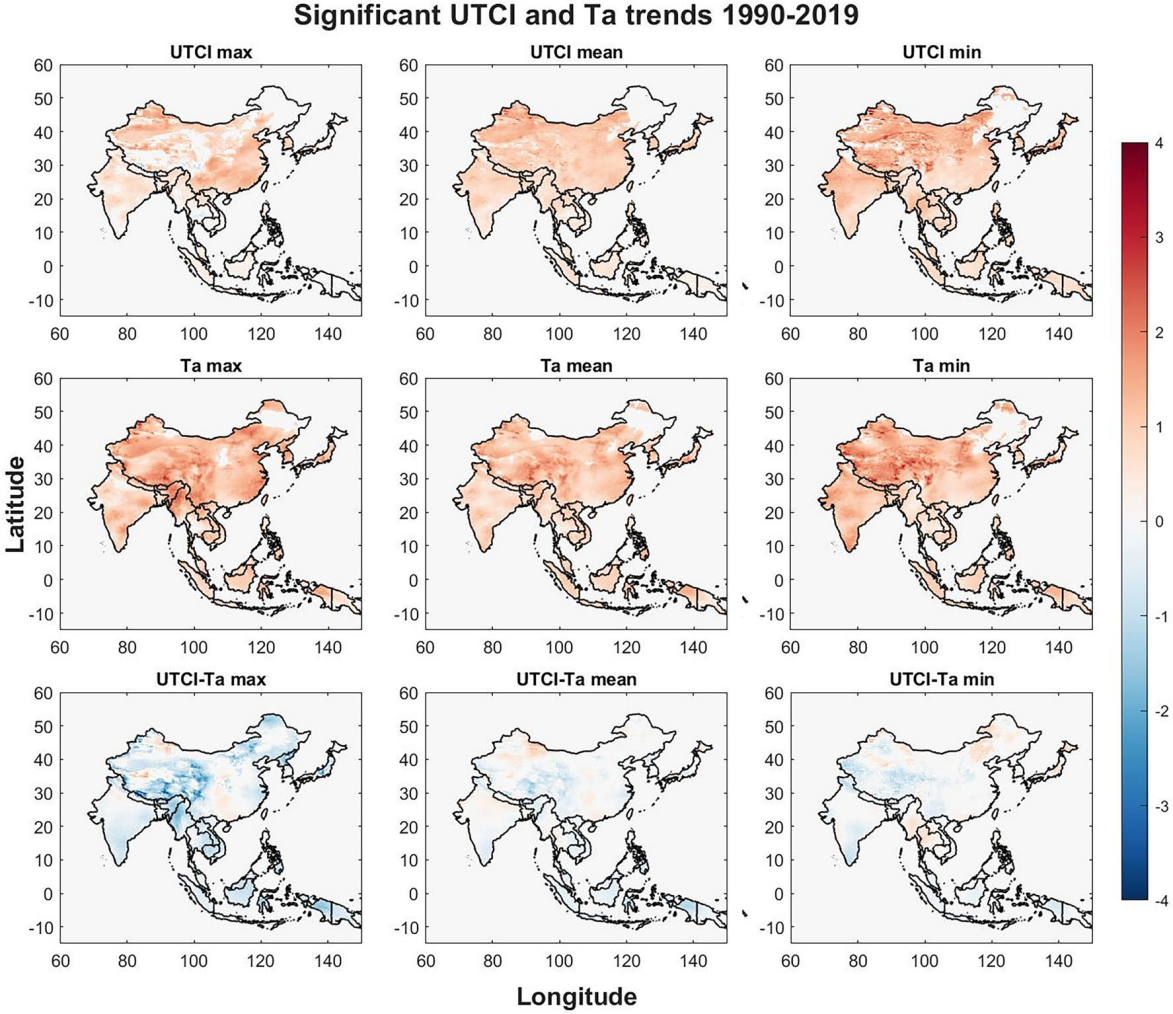
Significant trends can be seen for UTCI in the top row with UTCI max on the left, UTCI mean in the center and UTCI min on the right. The center row of plots represents Ta trends in the same order and bottom row the difference between UTCI and Ta for grid cells. The Figure was generated using MATLAB version 9.9.0 (R2020B), https://www.mathworks.com.

The spatial trends for UTCI and Ta are evaluated for the 30-year study period (1990–2019) in the Asia–Pacific region. Significant trends for UTCI are presented in Fig. 1, the trends are based on the slope of the trend and the color density corresponds to the absolute change in UTCI and Ta during the study period. Grid cells with insignificant trends and cells outside of the country borders are shown in white. The strongest increases in UTCI are found for UTCI min in central, eastern and northern China as well northern India, South Korea, Japan and the border between Myanmar and Thailand. The trends for UTCI mean are similar to those of UTCI yet slightly weaker and UTCI max only share the pattern in South Korea and for northern and eastern China while central China indicate both increasing and a few cells with decreasing trends. Southeastern China can be seen to have mild increase in UTCI min, moderate increase in UTCI mean while UTCI max show large increases for several regions. Increasing trends of Ta are more prominent in most regions where Ta min increase between 3 and 4 °C, primarily in central, eastern and northern China, Bhutan, southern and eastern India as well as southern Japan. Eastern Indonesia and northern Nepal shows moderate increase for all three Ta variables while northern Myanmar and the Manipur region of India show a strong increase for Ta max only. Cambodia, Laos, South Korea and North Korea show a greater increase for Ta max than Ta mean and Ta min. Almost all Ta trends increases in China except for parts of the northeastern provinces and parts of Guangxi and Guangdong provinces in the southeast where little to no significant increase is found.

The bottom row of Fig. 1 shows the difference between UTCI and Ta where most trends show that UTCI is lower than Ta. For max temperatures the biggest difference is found in eastern Tibet and the Tibet border to India and Nepal, Qinghai, and western Sichuan provinces in China. The border between the Manipur province and Myanmar, Mindanao in southern Philippines, central Honshu in Japan, North Korea, the island of Sulawesi in Indonesia and the Indonesian part of New Guinea show lower UTCI values. These patterns are weaker for mean temperatures, and most of the part of Tibet in China show lower UTCI values than Ta except for central western part of Tibet where UTCI is higher.

### Spatial analyses of moving delta trends

**Figure 2:**
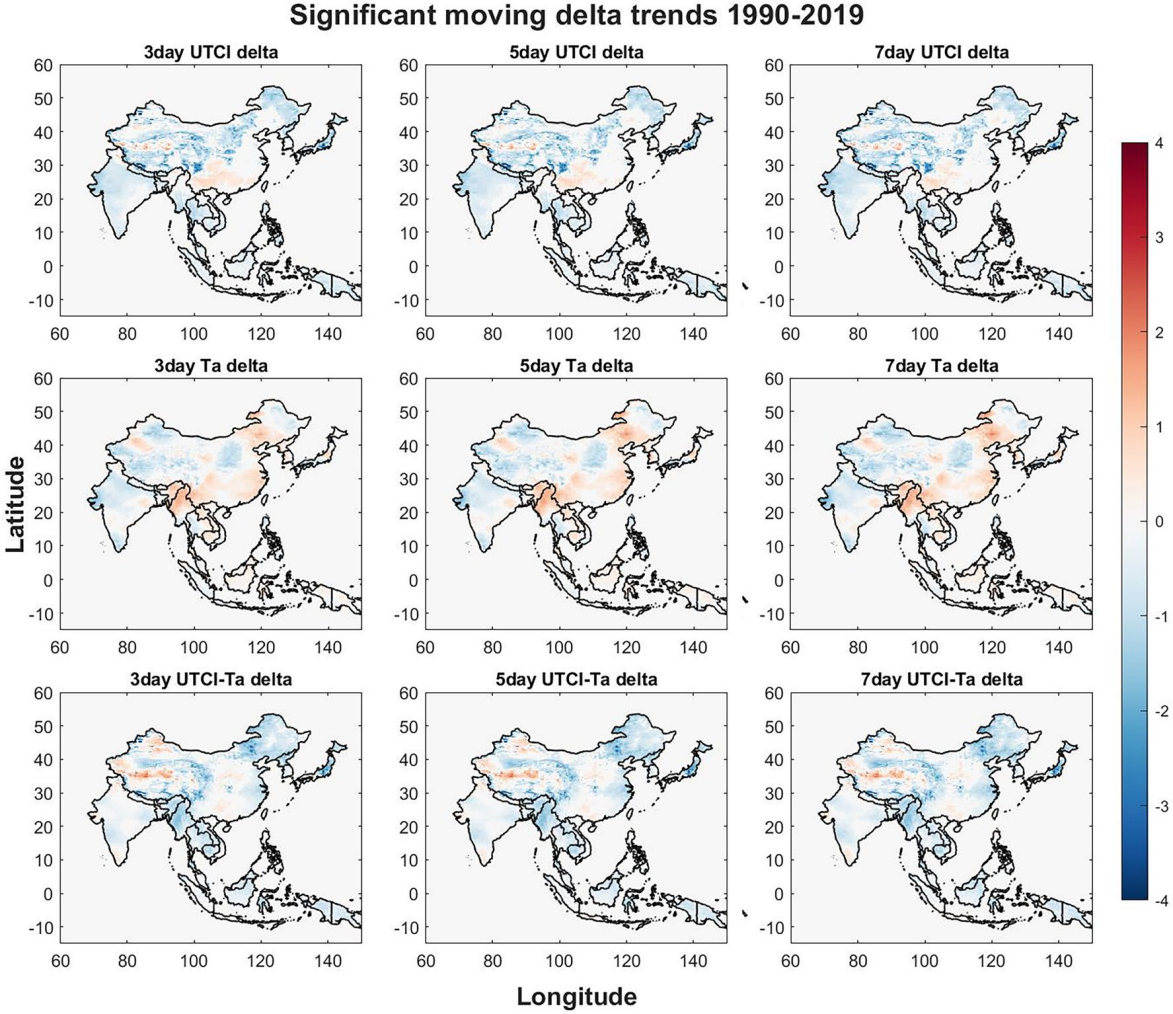
The moving delta for UTCI is shown on the top row, Ta on the middle row and the difference between UTCI and Ta on the bottom row. The 3-day moving window is the plot to the left, the 5-day moving window in the middle and 7-day moving window to the right. The Figure was generated using MATLAB version 9.9.0 (R2020B), https://www.mathworks.com.

The difference between the highest and lowest UTCI, Ta and difference between UTCI and Ta temperatures for a set window of days (3, 5 and 7 days) is visualized in Fig. 2 and is called moving delta trends from here on, the absolute change in temperature during the study period is presented on a color scale. The moving delta trends can provide insight if rapid changes in temperature over a few days are becoming increasingly prominent which may affect public health similar to the diurnal temperature range (DTR)^49^. The time window is set to cover short intense heat waves. The temporal patterns are very similar and only minimal differences can be seen between them. The border between Tibet and Xinjiang in China as well as southeastern China show an increase in the moving delta while the rest of China excluding the eastern provinces show a decrease. A decrease is also found in Japan, North Korea, western India, northwestern Nepal, northern Thailand and New Guinea. The moving delta for Ta shows a strong increase in northern Myanmar, the Manipur Province, and the eastern part of the Inner Mongolia and weaker increase in southeastern China. Most of India and the rest of China show decreasing trends. When comparing the significant difference between UTCI and Ta for the moving delta trend, several strong differences are found such as northern Tibet show higher increases in UTCI than Ta while most of China show lower UTCI than Ta. Northern Honshu and Hokkaido in Japan show lower UTCI than Ta which is also seen in North Korea, Myanmar, Manipur province in India, southern Thailand, Laos, Cambodia and New Guinea.

### Episodic difference analyses

**Figure 3:**
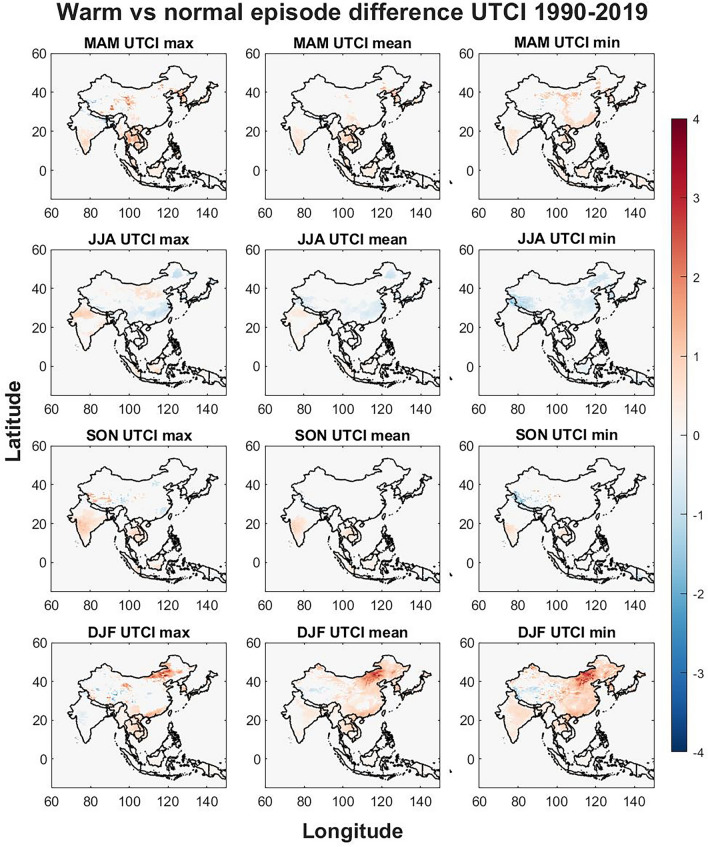
The warm vs normal episode difference is displayed for max, mean and min UTCI values for each season. The Figure was generated using MATLAB version 9.9.0 (R2020B), https://www.mathworks.com.

**Figure 4:**
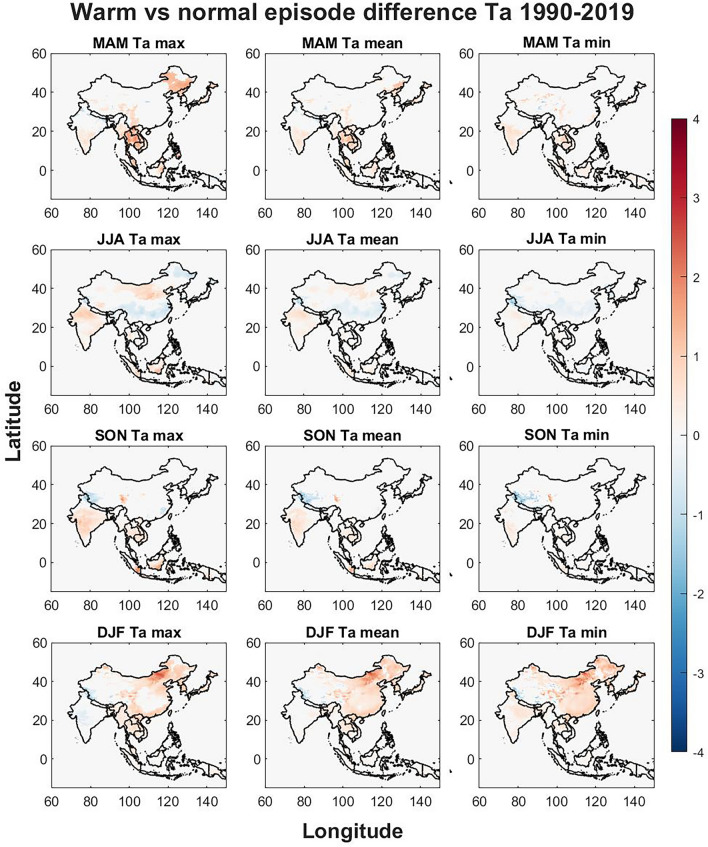
The differences in Ta between warm and normal episodes. The Figure was generated using MATLAB version 9.9.0 (R2020B), https://www.mathworks.com.

**Figure 5:**
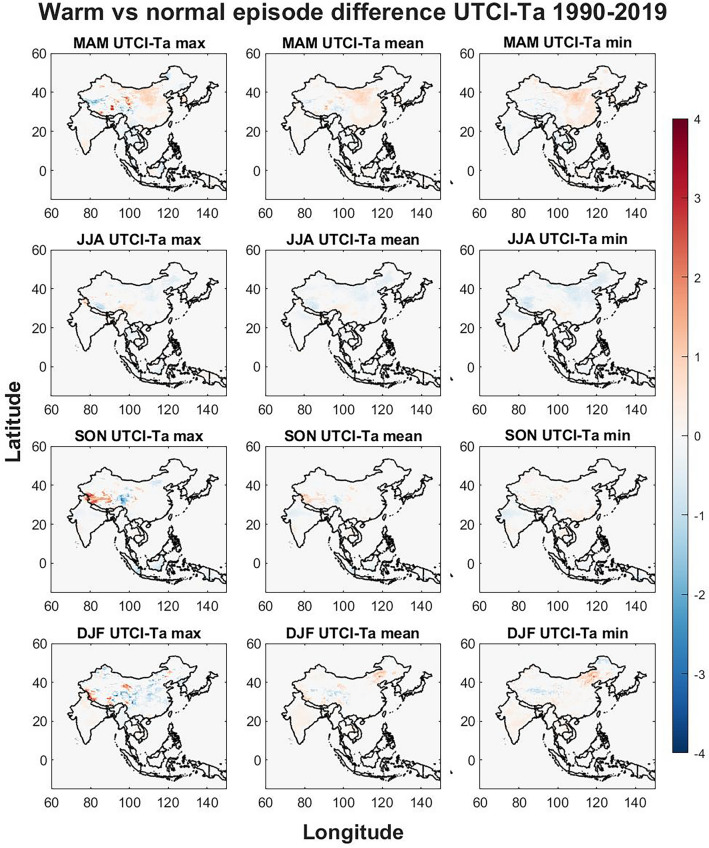
The seasonal differences between UTCI and Ta when comparing warm and normal episodes. The Figure was generated using MATLAB version 9.9.0 (R2020B), https://www.mathworks.com.

**Figure 6:**
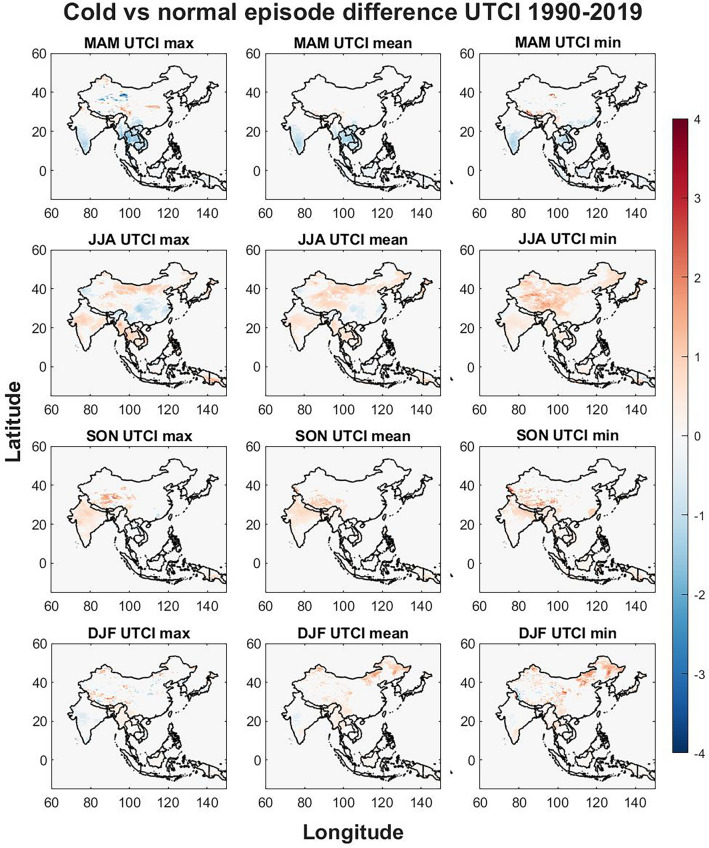
The seasonal differences in UTCI between cold and normal episodes. The Figure was generated using MATLAB version 9.9.0 (R2020B), https://www.mathworks.com.

**Figure 7:**
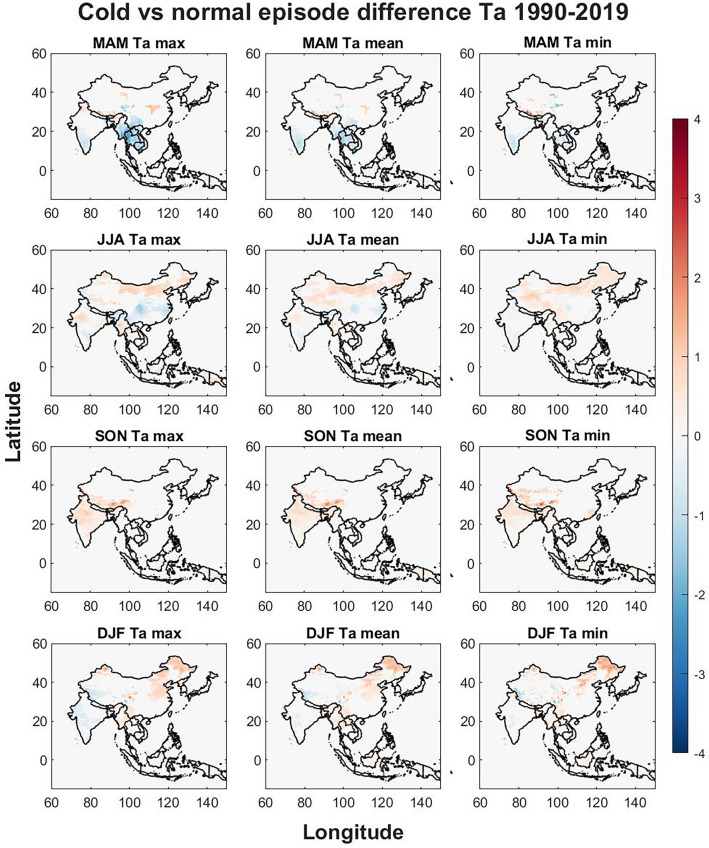
The seasonal differences in Ta between cold and normal episodes. The Figure was generated using MATLAB version 9.9.0 (R2020B), https://www.mathworks.com.

**Figure 8:**
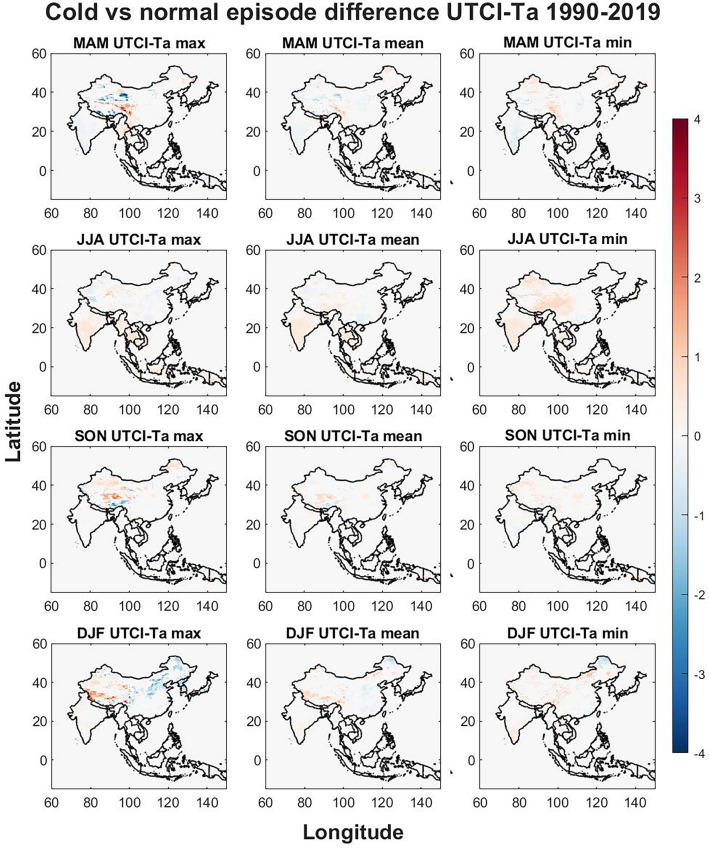
The seasonal differences between UTCI and Ta when comparing cold and normal episodes. The Figure was generated using MATLAB version 9.9.0 (R2020B), https://www.mathworks.com.

Seasonal differences of the warm and cold ENSO phase were compared to the normal ENSO phase to identify regional responses that may elevate public health risks. The seasonal differences are visualized in Figs. 3, 4, 5, 6, 7, 8 respectively for warm vs normal (UTCI, Ta, UTCI-Ta) and cold vs normal (UTCI, Ta, UTCI-Ta) for the study period over four seasons (MAM, JJA, SON and DJF). The colored grid cells in Figs. 3, 4, 5, 6, 7, 8 represent the difference in means between warm/cold and normal episodes. Only significant data are visualized using color density, non-significant data and grid cells outside of the studied countries are kept white.

#### Warm phase El Niño

The warm phase (Figs. 3, 4, 5) brings seasonal variations for UTCI (Fig. 3) where UTCI max show hotspots in central China and eastern Tibet in MAM, North Korea, South Korea, Cambodia, Laos, Vietnam and, southern India and most of Thailand also show an increase in UTCI max. The border of India to Nepal and Bangladesh shows a decrease in UTCI max including northern Bangladesh in MAM. UTCI mean is also increasing for warm phases in eastern Thailand, North Korea and South Korea while UTCI min has some increasing trends in northern and southeastern coast in China during MAM.

Southeastern China show a slight decrease for UTCI max in JJA while northern India show increasing trends. Southern South Korea, Hokkaido in Japan and the southern Japan show a decrease in UTCI max and mean while South Korea and northernmost part of India, western Nepal, and the western part of Tibet in China show a decrease during JJA for UTCI min.

SON show hotspots of strong increase in western and central Tibet and hotspots of strong decrease in eastern Tibet for UTCI max. Central India show increasing trends of UTCI max during SON which are weaker for UTCI mean and UTCI min. Decreasing trends are seen for UTCI min in western Tibet on the border to India.

DJF show several strong signals for UTCI max with increasing trends for northeastern and southern China, the Chinese border to India, central China, southern Laos, and South Korea. Hotspots of weakening trends for UTCI max are found in eastern Tibet. For UTCI mean and min during DJF, increasing trends are found in northern Vietnam, northeastern, northwestern and southern China, western Tibet, North Korea, and South Korea while a weakening trend is seen in northern and western Tibet. The greatest increase in UTCI is seen in central inner Mongolia province which is strongest for UTCI min during DJF.

The warm phase difference in Ta (Fig. 4) shows similar patterns to UTCI while some regions show different trends. For Ta max in MAM a strong increasing trend is found in northwestern China while the increase in UTCI in eastern Tibet are not seen in Ta. In JJA, a decreasing trend is found in northernmost India and southern China for all Ta variables while northern China show increasing trends for Ta max and similar for Ta mean albeit weaker. In SON a strong increase of Ta is found in Indonesia for the southern coast of Sumatra and the southeastern coast of Borneo for Ta max. A decreasing trend is found in western Tibet and a hotspot of increasing trends in eastern Tibet for all Ta variables while the trend is similar for UTCI min, it is reversed for UTCI max (Fig. 3). For DJF the Ta trends are similar to UTCI except for eastern Tibet where an increase is found for UTCI max while UTCI mean, UTCI min and all Ta variables show a decrease.

When comparing the significant difference between the UTCI and Ta trend (Fig. 5), there are notable spatial differences. Tibet show hotspots of both increasing and decreasing trends in MAM. Increasing UTCI trends compared to Ta for max, mean and min are seen in Northern and Southeastern China.

During JJA most trends indicate slight decreases in most of China. In SON for max temperatures there is a divide in Tibet where western Tibet shows increasing UTCI, and eastern Tibet shows decreasing UTCI compared to Ta. During DJF the strongest signals are found in China where Tibet show similar patterns to SON with an increase in the west and a decrease in the east with some increasing trends surrounding the eastern Tibetan plateau. UTCI trends are also higher in central inner Mongolia province in China compared to Ta.

#### Cold phase La Niña

The seasonal difference between the cold phase and normal phase is visualized in Figs. 6, 7, 8 for UTCI (Fig. 6), Ta (Fig. 7) and the difference between UTCI and Ta (Fig. 8). For MAM there are decreasing trends in southern India, mainland Thailand, southern Laos, Vietnam, Cambodia and slight decrease in Indonesia for all three UTCI variables (max, mean, min). For UTCI min there is a strong increase in southern Tibet and a decrease in southern China. For UTCI mean and max the decrease is found also in northern Laos and most of Myanmar while for UTCI max there are decreasing trends on the southern border of Tibet, increasing trends in eastern Tibet and decreasing trends north of Tibet in China.

For JJA there are mostly increasing trends in the study region. Northern China is increasing for all UTCI variables while for UTCI min most of China is increasing with the strongest signal in Tibet. Southern Guinea island and central Myanmar are increasing the most for UTCI max in JJA. Hokkaido and northern Honshu increase for all UTCI variables. A decreasing trend in JJA is found in the Manipur province and northern Myanmar, southeastern and western edge of China, Malaysia, and Brunei for UTCI mean and max. A decreasing trend is found in northern island of Guinea for UTCI min in JJA.

In SON, the strongest difference is found in Tibet where a bigger part is increasing for all UTCI variables. Parts of India are increasing in UTCI max and mean while only the northern part increase in UTCI min. Some decreasing trends are found in northern Thailand.

In DJF, most significant trends are found in China where increasing trends are seen in northwestern China for UTCI mean, and the increase is stronger and cover a larger part of northeastern China for UTCI min. Western Tibet show decreasing UTCI min in DJF while there are increasing and decreasing signals around eastern Tibet.

The cold phase differences for Ta (Fig. 7) are similar to those of UTCI (Fig. 6) with a strong decrease in the southern mainland countries during MAM, Tibet however shows a decrease in the westernmost part where UTCI is increasing and UTCI is decreasing in northern Tibet which is not seen in Ta but an increase is found on the border to Nepal. Southern India shows decreasing trends during MAM for all Ta variables. JJA trends for Ta show similar to UTCI where increasing trends are found in most of China except for southeastern China where decreasing trends are found which are most pronounced for Ta max. The patterns in SON for all Ta variables show increasing trends in southern Tibet and weak increasing trends for India. DJF show weak decreasing trends in India and western Tibet for Ta max and northwestern China and northeastern China show increasing trends for all Ta variables.

Figure 8 displays the difference between UTCI and Ta for cold phases and for MAM largest difference is found for max values where eastern Tibet show higher UTCI values while the border to Nepal, Bhutan and the Manipur province show lower UTCI compared to Ta which is also seen north of Tibet and Xinjiang. The pattern is similar for mean values, but the difference is smaller. In central China the UTCI is slightly higher than Ta for min values in MAM. In JJA UTCI is slightly higher than Ta for all variables for most regions except for the western part of China where UTCI trends are slightly higher than Ta for max temperatures. Southeastern Tibet in SON shows lower UTCI trends compared to Ta while northern Tibet shows a higher UTCI for all variables. In DJF Tibet show much higher UTCI for max temperatures while west of Tibet, northeastern China, North Korea, South Korea and central Honshu in Japan show lower UTCI max compared to Ta max.

## Discussion

The spatial heat pattern regarding climate change in Asia–Pacific region is revealed in this study where human thermal exposure is emphasized by including UTCI in the analysis. We can see that even though Ta is increasing (Fig. 1 middle row), UTCI (Fig. 1 top row) is not increasing at the same rate and shows different spatial patterns which is further evaluated when analyzing the difference between UTCI and Ta (Fig. 1 bottom row). The significant regions are found in the Manipur province in India, southern Tibet, northern Myanmar, and the island of New Guinea for max temperatures where UTCI is rather stable while Ta is increasing. The Himalayas and the Tibetan plateau stand out when comparing UTCI to Ta and may be because the region is still primarily experiencing cold stress which may be alleviated by the increasing temperature as found by Li and Chi^50^. As climate change may increase extreme weather and uneven heating, increasing wind speed especially in high altitudes may increase convective cooling which decreases UTCI. Even though UTCI increase is lower than Ta, UTCI is still increasing for most parts of the study region indicating an increase in heat stress risk. The results from this study align well with previous research; northwestern part of India has the highest increase of UTCI in the country^51^, the strong increase for Ta in Thailand during El Niño in spring season^52^, decreasing Ta in summer and increasing Ta in winter for El Niño in South Korea^53^. Ta shows similar spatial patterns with increasing trends for max, mean and min values except for the Manipur province and northern Myanmar which is mainly increasing for Ta max while southern India is increasing mainly for Ta min. These strong increases which are close to 3–4 °C would be associated with a great increase in heat exposure but this study highlights that the heat stress in parts of these regions is alleviated by other factors such as possible lower radiant temperature (e.g. more cloudy and rainy days), and/or higher air velocity. An increase of precipitation and number of wet days are found during the study period^39^ which could explain the alleviated heat stress. These results indicate that Ta is more sensitive to climate change compared to UTCI whereas UTCI is more relevant to human body heat exchange with the environment indicating the risk of relying on Ta for thermal stress analysis.

The moving delta trend analyses (Fig. 2) was performed to evaluate potential increases in health risks associated with DTR^49^. The window for UTCI is mainly decreasing which would indicate more even thermal stress distribution with less relief during nights throughout the days leading to more long-term thermal stress risks. The moving trend for Ta is slightly different with more increasing deltas compared to UTCI. The biggest difference is found in northern Myanmar, the Manipur province, and eastern Inner Mongolia where the delta for Ta is increasing indicating more erratic temperature profiles which could impose more short-term thermal stress risks. When comparing the UTCI moving delta to the Ta moving delta, the biggest increasing difference is found in northern Tibet. Decreasing differences are found in southern Tibet, Qinghai, western Sichuan, eastern Inner Mongolia, Honshu in Japan, Myanmar, Cambodia, and eastern North Korea.

By evaluating the results presented in Figs. 1 and 2 we can state that UTCI is steadily increasing and particularly in the northern part of the study region, the UTCI moving delta for the same region is decreasing which indicates that the thermal stress risk in this region will increase in both intensity and duration as the values increase while the recuperation periods during the colder parts of the days are also becoming increasingly thermally stressful. The increase of UTCI may shift the categorized level of heat stress observed in several regions as both moderate and strong UTCI heat stress levels are set using 6 °C ranges^18^ and observed increases in Fig. 1 are close to 2–3 °C making a shift from moderate to strong or even from strong to very strong heat stress likely.

The effects of ENSO on seasonal temperatures were evaluated in Figs. 3, 4, 5, 6, 7, 8 for both warm episodes (El Niño) and cold episodes (La Niña) compared to the normal state. The greatest increase was found during DJF where El Niño triggers increasing UTCI and Ta (Fig. 3) and Ta (Fig. 4) temperatures, particularly western Inner Mongolia. The greatest decrease is found in MAM where La Niña affects UTCI (Fig. 6) and Ta (Fig. 7), particularly in the region around northern Thailand.

Northern Thailand has an increase during MAM for max UTCI and Ta values during warm episodes while southern Sumatra and Borneo has an increase during SON for Ta max which is not seen for UTCI. The Himalayas both on the Tibet and Nepal side has a decrease in UTCI and Ta during warm episodes, particularly in SON, except for UTCI max which increases. Southeastern China and Hokkaido in northern Japan sees a decrease in UTCI and Ta during JJA during warm episodes. The warm episodes appear to increase UTCI and Ta for MAM and DJF while JJA show more decreasing trends. Cold episodes greatly decrease UTCI and Ta in the southern half of the study region during MAM while JJA brings a weak increase for UTCI and Ta to most of the study region except for southeastern China that show decreasing trends. The Himalayas and Tibet show an increase in both UTCI and Ta during SON for cold episodes and Northeastern China seems to get warmer with increasing heat during DJF.

The spatial differences indicate the importance of the regional topography and relation to the seasonal weather systems. The Himalaya with its great elevation appears to be particularly exposed to climate change with increasing UTCI and Ta and greatly affected by ENSO. Yet it is worthwhile to point out that even though Ta appears to increase greatly in southern Tibet, UTCI is not increasing as rapidly. Ta increases in almost all parts of the study region while the southern half shows only a weak increase, yet UTCI indicates that the risks of thermal stress will increase moderately indicating that changes in humidity, air velocity, and/or radiant temperature and precipitation may not be consistent with air temperature changes^39^. The greatest significant changes brought by ENSO appears to be increasing UTCI and Ta in western Inner Mongolia in China during winter for warm episodes and similar tendencies during cold episodes. The south coast in China increase in UTCI and Ta during winter for warm episodes. Warm episodes also increase UTCI and Ta max in Northern Thailand during spring while cold episodes show a great decrease for Thailand and its neighboring countries for both UTCI and Ta in spring. Tibet in particular show spatial variations during autumn where for western Tibet an increase for UTCI max and a decrease in eastern Tibet while this is reversed for UTCI min and the Ta values during warm episodes. Cold episodes bring a more uniform increase to UTCI and TA in southern Tibet during autumn.

Better understanding climate change patterns are very important for adaptation actions, public health resilience and worker health. Productivity is known to be greatly affected by heat stress^11^ and changes in climate affect vector-^54^ and waterborne diseases^55^ such as diarrhea. The Asia–Pacific region is identified to be vulnerable to climate change where ENSO further leads to increased vulnerability due to large precipitationvariability^56^. Heat-health early warning systems are becoming more commonly utilized^57^ and the further integration of human thermal models that predict thermal stress provide more relevant information for dangerous heat exposure and heat exchange than Ta alone^58^. By further including ENSO and other teleconnections into early warning systems on sub-seasonal to seasonal timescales to predict health and disease complications, negative climate change impacts can be reduced, and societal resilience may be improved. This study suggests that the simplicity of Ta can be misleading regarding human thermal stress as it depends not only on air temperature but also other thermal climatic variable and personal factors such as clothing and activity. UTCI with all these relevant variables integrated can complement the limitation of Ta to predict heat impact on health.

This study evaluates the change of UTCI and Ta based on reanalysis data which introduce uncertainties compared to measured weather station data due to the complexity to model climate data^35^. The findings of this study would benefit by further evaluating the region using measured data and compare it with the reanalysis data. In this study, only the Ta component of UTCI is evaluated and differences are found. To determine the local dominant climate factor of heat stress, all variables related to heat transfer between the human body and the environment should be evaluated. The universal aspect of UTCI could also be evaluated for different populations to assess if long-term adaptation impacts UTCI thresholds and evaluate multiple UTCI thresholds for vulnerable groups. The effect of altitude was not evaluated in this study, this should be further analyzed as the findings in this study suggest that Tibet experiences a greater rate of change than many other regions. The ERA reanalysis data has been identified as the most appropriate reanalysis model when assessing the temperature in the region^59,60^ but the complex terrain calls for a cautious approach and more research is suggested to accurately quantify the thermal stress. In this study, only the direct association of ENSO was evaluated, and further analysis of lag effects could provide a better understanding of how ENSO affects thermal stress in the region.

## Methods

The UTCI and Ta data were processed and analyzed using MATLAB R2020b. Figures 1, 2, 3, 4, 5, 6, 7, 8 were generated in MATLAB R2020b using the add-on ColorBrewer to set the color gradient^61^. The study region was defined as 60°N to 15°S and 60°E to 150°E. The study period is 1990–2019. Grid cells with less than 70% area inside the country border were excluded from the analyses. The spatial resolution of UTCI and Ta gridding data is 0.25°. The temporal resolution of the raw data for UTCI and Ta was hourly, and the analyses were performed on daily max (95th percentile) mean and min (5th percentile) values. When evaluating the difference between UTCI and Ta, the significance test was performed on the resultant. The trends for UTCI and Ta were tested using the non-parametric Mann–Kendall’s trend test which evaluates there is trend is upward or downward by ranking the observations^62,63^. The statistical significance level in this study was set at 0.05. The slope of the significant trends is given by a linear regression model and the total change in temperature between 1990 and 2019 is visualized in Figs. 1, 2, 3, 4, 5, 6, 7, 8 where the total change is the slope value times the timestep.

UTCI is calculated using a 6th order polynomial regression function and can be calculated on www.utci.org which also has Fortran codes available. UTCI is defined as the temperature of the reference condition which causes the same physiological reactions as the condition of interest, where the UTCI deviates from Ta based on the humidity, wind speed and mean radiant temperature. The reference condition assumes that individuals maintain a set metabolic rate corresponding to a 4 km h^−1^ walk wearing clothing insulation that is calculated based on the Ta ^19,64^ in an environment of 0.5 m s^−1^ wind speed at 10 m height, mean radiant temperature equal to Ta and relative humidity at 50% except for high temperatures (> 29 °C) where water vapor pressure is set to 20 hPa.

The moving delta analysis is evaluated using different number of days for the window of interest. For the 3-day moving window, the highest UTCI and the lowest UTCI value found during day 1–3 is the first delta value, the second delta value is calculated using day 2–4 and so on. Similar to Fig. 1, the total change in delta temperature is visualized in Fig. 2.

The equality between ENSO episodes is tested using a Wilcoxon rank-sum test^65^. The Wilcoxon rank-sum test is also known as the Mann–Whitney U which is a non-parametric statistical test to test the equality of the medians for two populations of two independent samples. The null hypothesis is that the medians of the two samples are equal. The level of significance was set to 0.05. The data was saved on a seasonal level and classified as either warm, normal, or cold according to the National Oceanic and Atmospheric Administration (NOAA) Niño 3.4 SST index where warm/cold episodes are defined as one standard deviation above/below the seasonal SST anomaly in the Niño 3.4 area. The warm, normal, and cold seasons are listed in Supplementary Table 1. The cells with significant differences are compared in Figs. 3, 4, 5, 6, 7, 8 where the mean is compared between the episodes.

## Conclusions

The increasing temperatures in Asia–Pacific region followed by climate change are further analyzed and visualized in this study with emphasis on extreme heat using UTCI and Ta. The link between ENSO and heat stress is evaluated using UTCI and it is found to be increasing but at a lower rate than Ta during 1990–2019. The difference between the highest and lowest UTCI recorded in a 3-day, 5-day and 7-day time window is decreasing with time which is also true for Ta which may increase the long-term thermal stress. The seasonal effects of ENSO are evaluated, and several regions such as Tibet, eastern Inner Mongolia in China and Thailand appear more influenced than others. By further developing early warning systems using thermal stress indices and by including teleconnections such as ENSO, sub-seasonal to seasonal warning systems may improve the resilience of the region.

### Supplementary Information

Supplementary Information.

## Data Availability

The UTCI and Ta data used in the study was retrieved from the Copernicus Climate Change Service C3S by ECMWF^66^. ERA5 and ERA5-HEAT data are generated using a parameterization scheme at a high spatiotemporal resolution and an advanced assimilation system. The ENSO index Niño 3.4 SST is available from the NOAA and the index is calculated using the HadISST1 dataset. The MATLAB code is available upon request from the corresponding author.
