# Supplementary material for: Spatiotemporal link between El Niño Southern Oscillation (ENSO), extreme heat, and thermal stress in the Asia–Pacific region
Source: Sci Rep. 2024 Mar 28;14:7448. doi: 10.1038/s41598-024-58288-0 (PMC10978954; doi:10.1038/s41598-024-58288-0)
Supplement: Supplementary file 1 — Supplementary Information. [file 41598_2024_58288_MOESM1_ESM.docx]

Supplementary information for

**Spatiotemporal link between El Niño Southern Oscillation (ENSO), extreme heat, and thermal stress in the Asia-Pacific region**

Jakob Eggeling^1^, Chuansi Gao^1^, Dong An^2^, Raul Cruz-Cano^3^, Hao He^4^, Linus Zhang^2^, Yu-Chun Wang^5^, Amir Sapkota^6^

^1^Aerosol and Climate Laboratory, Division of Ergonomics and Aerosol Technology, Department of Design Sciences, Faculty of Engineering (LTH), Lund University, Sweden

^2^Division of Water Resources Engineering, Faculty of Engineering (LTH), Lund University, Sweden

^3^Department of Epidemiology and Biostatistics, Indiana University, School of Public Health, Bloomington, IN 47405, United States

^4^Department of Atmospheric and Oceanic Science, University of Maryland, College Park, MD 20742, United States

^5^Department of Environmental Engineering, College of Engineering, Chung Yuan Christian University, 200 Chung-Pei Road, Zhongli 320, Taiwan

^6^Department of Epidemiology and Biostatistics, University of Maryland, School of Public Health, College Park, MD 20742, United States

Supplementary Table 1. The seasons are classified as warm, normal, or cold based on the Niño 3.4 SST index and are listed in the corresponding column below.

|  | **MAM** | | | **JJA** | | | **SON** | | | | **DJF** | | | |
| --- | --- | --- | --- | --- | --- | --- | --- | --- | --- | --- | --- | --- | --- | --- |
|  | 1992, | 1993, | 1998, | 1991, | 1997, | 2002, | 1991, | 1994, | 1997, | 2002, | 1991, | 1994, | 1997, | 2002, |
| Warm episodes | 2015, | 2016, | 2019 | 2009, | 2015 |  | 2004, | 2006, | 2009, | 2014, | 2004, | 2006, | 2009, | 2014, |
|  |  |  |  |  |  |  | 2015, | 2018 |  |  | 2015, | 2018, | 2019 |  |
|  | 1999, | 2000, | 2008, | 1998, | 1999, | 2000, | 1995, | 1998, | 1999, | 2000, | 1995, | 1998, | 1999, | 2000, |
| Cold episodes | 2011 |  |  | 2010 |  |  | 2007, | 2010, | 2011, | 2016, | 2005, | 2007, | 2008, | 2010, |
|  |  |  |  |  |  |  | 2017 |  |  |  | 2011, | 2017 |  |  |
